# A Word-Granular Adversarial Attacks Framework for Causal Event Extraction

**DOI:** 10.3390/e24020169

**Published:** 2022-01-24

**Authors:** Yu Zhao, Wanli Zuo, Shining Liang, Xiaosong Yuan, Yijia Zhang, Xianglin Zuo

**Affiliations:** 1 Colledge of Computer Science and Technology, Jilin University, Changchun 130015, China; yuzhao19@mails.jlu.edu.cn (Y.Z.); wanli@jlu.edu.cn (W.Z.); liangsn19@mails.jlu.edu.cn (S.L.); yuanxs19@mails.jlu.edu.cn (X.Y.); yijia18@mails.jlu.edu.cn (Y.Z.); 2 Key Laboratory of Symbolic Computation and Knowledge Engineering of Ministry of Education, Jilin University, Changchun 130015, China

**Keywords:** causal event extraction, reinforcement learning, adversarial attack, information extraction

## Abstract

As a data augmentation method, masking word is commonly used in many natural language processing tasks. However, most mask methods are based on rules and are not related to downstream tasks. In this paper, we propose a novel masking word generator, named Actor-Critic Mask Model (ACMM), which can adaptively adjust the mask strategy according to the performance of downstream tasks. In order to demonstrate the effectiveness of the method, we conducted experiments on two causal event extraction datasets. Experiment results show that, compared with various rule-based masking methods, the masked sentences generated by our proposed method can significantly enhance the generalization of the model and improve the model performance.

## 1. Introduction

Constructing a causal knowledge graph has essential impact on natural language reasoning tasks. In addition, how to extract causal event pairs from texts is a fundamental problem. In the early studies, causal event extraction was formulated as a pipeline process [1]. It identifies all potential event pairs and performs causal relation classification for each of them. However, this extraction method tends to suffer from the error propagation problem, which means the error from the extracted event pair will be passed to the classification process. One way to mitigate this effect is a sort of sequence labeling task [2]. The task performs a multi-class identification on each word in sentence. Through such a sequence labeling task, the problem in the pipeline method can be solved. In general, this joint sequence labeling method focuses on how to obtain word semantic representations to achieve better results.

With the emergence of large scale pre-trained language models [3,4,5], multiple downstream tasks have achieved SOTA results. In our experiments, the representation of pre-trained language models is also suitable for causal event extraction task. On the other hand, the current causal event extraction datasets are relatively small. A language model, such as BERT, has very large amount of parameters. It is unsuitable to add more components to the model, which is very prone to overfitting problems. With this in mind, data augmentation on a small corpus is an effective method.

**Figure 1:**
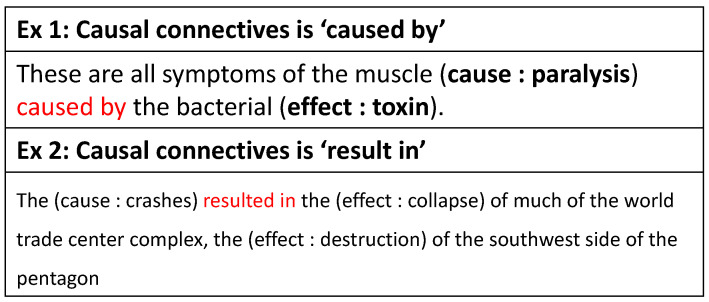
Examples of sentences with pairs of causal events.

For causality extraction, the target is to extract event pairs with causal relations. Some sentences contain explicit causal connectives, such as “*cause*” and “*due to*”. These connectives clearly show the causal relationship between events. Most of the causal event pairs are on the left and right sides of the connective, as shown in the Figure 1. Furthermore, there are some sentences that contain causal events with no obvious connectives. To identify implicit causal relation, we need to pay more attention to the semantic connection between causal event pairs rather than connectives. How to identify event pairs in implicit causal relationships is a difficult problem. In this paper, our model uses adaptive masking of causal connectives to make the model more focused on the relationship between events. This method allows the model to extract events that have causal relationships without excessive causal conjunctions.

On the contrary, the original method of masking word is a random process. The performance depends on multiple random selections, which is unstable. In this paper, we propose a mask word generation model based on reinforcement learning. It generates a task-adaptive mask word distribution through confrontation with the BERT model, which can mask words in a targeted manner to capture the semantic relation of causal events better.

Specifically, we adopt the A2C framework to obtain a mask word distribution generator. It consists of a BERT-based encoder, a relational classifier and a mask position generator. Experiments show that, for the two datasets, the performance of using a large pre-training model has surpassed the SOTA model of the sequence labeling task. However, a model with adaptive mask generator can achieve a better performance. At the same time, we also compared with the rule-based method, and it also shows the superiority of our model.

The main contributions of our work are as follows:

- We propose an adaptive mask model that utilizing reinforcement learning to obtain a word mask distribution. In addition, through adversarial training methods, the generalization performance of large models on small datasets can be improved. Our method proposes a new perspective to combine reinforcement learning and deep learning to solve problems.
- We calibrate the causal dataset extracted from a benchmark dataset and obtain a more accurate one with causal event pair annotation.
- We conduct extensive experiments on the causal event extraction datasets. Experiments show that the model with ACMM is better than its basic version.

## 2. Related Works

### 2.1. Causality Extraction

As an essential task, the causality extraction task has applications in many upstream tasks, such as event prediction [6], scenario generation [7], question and answering [8], and so forth. In some real-world tasks, such as medicine [9], there are also applications of causality extraction. At a higher level, the task of causal extraction and causal reasoning is inextricably linked. In some viewpoints, artificial intelligence will be fundamentally restricted if there is no causal reasoning ability [10].

At present, the research on causality extraction based on deep learning is mainly divided into three categories. The first is to use the method of pattern matching, which uses semantic features and vocabulary symbol features to extract causality [11]. This method, that completely relies on pattern matching, has poor cross-domain adaptability and requires expert knowledge. The second method is based on a combination of pattern matching and machine learning [8,12]. This method generally uses a pipeline method. First, the pattern matching method is used to extract the potential causal entity pairs, and then the machine learning method is used to judge the candidate causal pairs. However, this method also requires domain knowledge and has the problem of error transmission, i.e., errors in the first stage will affect the effect of the second stage. The third method is based on deep learning. Because it does not require domain knowledge and can effectively capture ambiguous causality, it has become the most commonly used method in recent years. Some commonly used models have applications in causality extraction, such as CNN [13], LSTM [14], etc. At the same time, there are some studies on the introduction of external knowledge for causal extraction [15].

### 2.2. Pre-Trained Language Model

Using a representation language model pre-trained from large-scale unlabeled text is a universal and effective method in most natural language understanding tasks [16]. Most of these language models use self-supervised training methods. With the success of MLM pre-training [17], a large number of MLM-based pre-training methods and models have been proposed [4,18,19,20]. However, these tasks are limited to applying the mask strategy to the pre-training stage. There is no attempt to learn a mask strategy for downstream tasks and data, which is the problem we want to solve in this work.

### 2.3. Reinforcement Learning-Based NLP

Deep reinforcement learning is another system different from neural networks. It currently performs well on many decision-based problems. For example, in games or other fields, it has many applications [21,22,23]. At the same time, we believe that the application of reinforcement learning to NLP still has great potential. In some NLP tasks involving serialization decision-making, reinforcement learning has many applications, such as multi-document information extraction [24], reference resolution [25], text classification [26], text summarization [27], etc. In most NLP tasks, reinforcement learning is used as an auxiliary task. For example, reinforcement learning is used as a model for deleting irrelevant words and judging the structure of the current phrase in text classification tasks [26]. In addition, in text summarization, the reinforcement learning model distinguish important paragraphs from a large amount of text to filter irrelevant information. Then, the deep learning model uses the condensed text for text summarization tasks. RL method is also used in large-scale pre-training language models. For instance, a masking word strategy is learned through reinforcement learning to enhance the convergence rate [28] or task adaptation capabilities of the pre-training model [29]. The difference between this article and the above work is that we focus on the fine-tuning stage of the task and design a masking word strategy against the model to improve the generalization of the model.

## 3. Materials and Methods

### 3.1. Datasets

We evaluated our framework on the public datasets SemEval-2010 Task 8 [30] and Causal TB (Causal TimeBank) [31] (only the entity annotations for causality are extracted). We only kept the causal annotations for the Causal TB dataset and transformed them into a sequence annotation dataset. For the SemEval dataset, some parts of the causal event pair are not accurately labeled. Therefore, in order to identify the causal relationship, we performed the following processing on the dataset.

**Figure 2:**
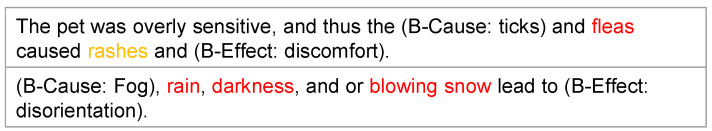
Examples of reprocessing data. The red and yellow words in the table are the newly added reason and result labels, respectively.

- Each word pair with a causal relationship is labeled as one of the “B-Cause”, “I-Cause”, “B-Effect”, “I-Effect”; other relationships and irrelevant words are labeled as “O”.
- The SemEval dataset only contains a pair of causal relationship entity tags. Yet, there is a variety of one-to-many, many-to-many causal relationships (Examples are shown in Figure 2). We have labeled these data.

The details of the two datasets are shown in the Table 1. After reprocessing, the causal event pair of the SemEval dataset is expanded from 904 to 1053. Due to the lack of datasets for causal event relations, our data volume is relatively small, which can illustrate the improved generalization performance of our adversarial model.

### 3.2. Methods

**Figure 3:**
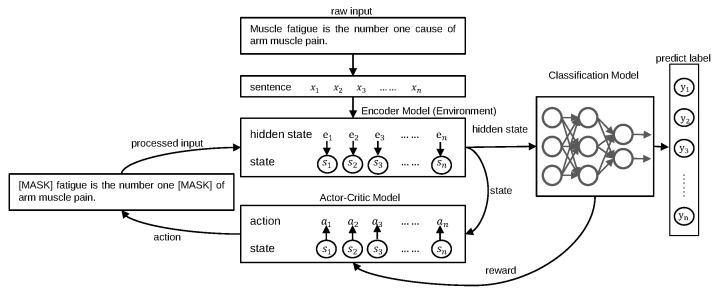
An overview of the proposed ACMM framework.

Our framework is mainly divided into two parts: language model warm-up; and mask model and language model confrontation training. In the initial stage, we use conventional methods to warm-up the language model, which means we fine-tune the model on the train dataset. After obtaining a better language model, we regard it as an environment model to warm-up the mask model. Finally, after obtaining a stable mask model, we use the adversarial training method to train two models simultaneously. The main structure of the frame is shown in Figure 3.

#### 3.2.1. Encoder Model Warm-Up

We employ pre-trained BERT as the primary language model and use the word-piece sequence after word segmentation as input to obtain a high-dimensional representation of the sentence. In subsequent training step, it is used as the original input into each model. Before introducing the model, let us briefly clarify the mathematical notations we used. We use $X=(x_{1},\ldots ,x_{n})$ to represent an input sample, where $x_{i}$ represents the index of each word. E represents the hidden state generated by the encoder model, where $E=(e_{1},\ldots ,e_{n})$. $\theta_{encoder}$ represents the parameters of the context encoder model used (BERT), $\theta_{cls}$ represents the parameters of the classifier model, and $\theta_{a2c}$ represents the parameters that mask word generator model used. The masked position generated by the masked word generator is replaced with [MASK].

First, we use the encoder model to obtain the hidden representation of the sentence and then obtain the classification of each word through the classifier. Then, we calculate the loss through the cross-entropy loss function (1).

(1) $$L_{cls}\left(\theta_{encoder},\;\theta_{cls}|X\right)=\;\frac{1}{N}\sum_{i=1}^{N}-\sum_{c=1}^{M}z_{j}^{c}\log\left(p_{j}^{label=c}\right),$$

(2) $$z_{j}^{c}=I\left(y_{j}^{c}=y_{j}^{label}\right).$$

*N* is the number of words. *M* is the number of tags, which is 5 in this article. $z_{j}^{c}$ is an indicator function. $z_{j}^{c}=1$ means the predicted label is equal to the actual label. In the warm-up stage, we use the BERT model and classifier to fine-tune the training set. In the experiment, we use 10 epochs for warm-up, which is a hyperparameter.

#### 3.2.2. Environment Model

For most reinforcement learning algorithms, we need to obtain the current state score and the next state. Both of them are taken from the environment. In order to make it suitable for the training of the mask model, we adopt the following method:

We utilize the high-dimensional vector obtained by the BERT encoder model to represent the state. For ease of use, we have performed dimension reduction operations on it.

(3) $$S=\;Encoder\left(X;\;\theta_{encoder}\right)W_{\mathrm{env}}+b_{\mathrm{env}}.$$

We use $X_{\mathrm{mask}}$ to represent the sequence masked by the mask model, and the next state is represented as:

(4) $$S_{next}=\;Encoder\left(X_{\mathrm{mask}};\;\theta_{encoder}\right)W_{\mathrm{env}}+b_{\mathrm{env}}.$$

For each state *S*, we regarded the loss produced by classifier as the score.

(5) $$score=L_{cls}\left(\;\theta_{encoder},\theta_{cls}\;|X\right).$$

#### 3.2.3. Mask Model Overview

For the mask model, we use the advantage-actor-critic [32] framework. Since this task cannot be modeled as a split-screen Markov process, we transform this task into a TD(0) method. Specifically, we use an actor model to generate the action distribution $p_{a}$ of current state. In order to obtain the more extensive exploration space of reinforcement learning, we sampled $p_{a}$ repeatedly and obtained multiple next states through different actions ${state}_{i}=\left({state}_{1},\ldots ,{state}_{n}\right)$. Through the environment model, we obtain the scores for these states, and then the reward of each action is calculated as the formula:

(6) $$r_{i}=\;ReLU\left(score\left({state}_{i}\right)\;/\;score\left(state\right)-1\right).$$

As the environment model is trained, its score will become smaller. To avoid the impact of the decreased score value, we use the form of percentage to represent the improvement of the score, which indicates how much the score has improved compared to the previous state. At the same time, we limit the number of covered words, which is a hyperparameter. It is obvious that the more words are covered, the vaster the losses are generated. All in all, Reward indicates that the model hopes to mask the word that has the most significant impact on predicting. Finally, we will obtain a mask distribution $p_{a}$ for the task. Subsequently, we will introduce the details of the actor model and the critic model, respectively.

#### 3.2.4. Critic Model

In the A2C framework, the critic model is generally used to calculate the state value. First, in order to make effective use of BERT’s powerful language representation capabilities, the critic model and the actor model share the dynamic word vector *E* generated by the BERT encoder, yet it does not perform backpropagation. In the low layer, the critic model uses a simple linear layer to estimate the current state’s value. Then, we employ the Mean Square Error function to calculate the difference between the estimated value and the reward.

(7) $$V_{\pi }\left(s\right)\;=P\left(\theta_{critic}|state\right),$$

(8) $$L_{critic}=MSE\left(R,V_{\pi }\left(s,\;a\right)\right).$$

#### 3.2.5. Actor Model

We use the advantage function to update the policy gradient of the actor model. Specifically, we use A to represent the advantage function, which able to reduce the gradient for stable training. The calculation of the advantage function is as follows:

(9) $$A_{\pi }\left(s_{t},a_{t}\right)\;=r\left(s_{t},a_{t}\right)+V_{\pi }\left(s_{t+1}\right)-V_{\pi }\left(s_{t}\right).$$

Therefore, using the policy gradient method [33], our gradient to be updated is

(10) $$\nabla J\left(\theta_{actor}\right)\;=\nabla_{\theta_{actor}}-\log\pi_{\theta_{actor}}\left(a_{t},s_{t}\right)A_{\pi }\left(s_{t},a_{t}\right).$$

Update the strategy parameters $\theta_{actor}$:

(11) $$\theta_{actor}=\theta_{actor}+\alpha \nabla J\left(\theta_{actor}\right).$$

The A2C model is not a method of calculating value functions, and its on-policy feature makes it impossible to use Experience Replay operations. For the purpose of making the model converge faster, we use a function similar to Experience Replay, which is different from the function in DQN. We store each (state,action,reward,next_state) quadruple in the reply buffer and, when the reply buffer is full, randomly select a batch of data for training. We do this because we need the predicted probability and its calculation graph corresponding to each action during training. It is unrealistic to save. However, our purpose is only to obtain a distribution that covers each word. To this end, we make use of each state to re-pass the model to obtain a calculation graph of the current strategy and then use the previously explored action to update the parameters. In this way, we can meet its on-policy property and explore a larger space for action.

### 3.3. Adversarial Training

In pace with training of environment, the scores corresponding to the same state will also change. Thence, after the hot start, we need to train the reinforcement learning part and the deep learning part at the same time. We adopt a kind of adversarial training method, and the details as shown in Algorithm 1.
**Algorithm 1** Adversarial training.**Require:** environment model ENV, mask model A2C, dataset *D*, Sampling threshold ϵ
1:Fixed environment model parameters $\theta_{encoder}$, $\theta_{cls}$;2:**while** done **do**3:   **for** data in *D* **do**4:     Train the mask model and update the parameters $\theta_{a2c}$;5:   **end for**6:   fixed mask model parameters $\theta_{a2c}$7:   **for** data in *D* **do**8:     Sampling random number $r\in \left[0,\;1\right]$;9:     **if** r>ϵ **then**10:        Get new data ndata from A2C model;11:        Train the ENV model and update the parameters $\theta_{encoder}$, $\theta_{cls}$ with ndata;12:     **else**13:        Train the ENV model and update the parameters $\theta_{encoder}$, $\theta_{cls}$ with data;14:     **end if**15:   **end for**16:**end while**


## 4. Results and Discussion

### 4.1. Experimental Setting

#### 4.1.1. Evaluation method

In this part, we will introduce our evaluation indicators: P (Precision), R (Recall), and F1 (F1-score). Their calculation formula is as follows:

(12) $$P=\frac{TP}{TP+FP},R=\frac{TP}{TP+FN},F1=\frac{2\times P\times R}{P+R}.$$

Among them, *TP* and *FP* indicate how many labels predicted to be Positive are correct and incorrect, respectively. *FN* means the number of labels predicted to be negative is actually wrong. In short, *P* represents how many of the predicted true labels are truly true labels, and *R* represents how many truly true labels are predicted.

For this sequence labeling task, the number of “O” labels is far greater than the number of causal labels. Therefore, we first calculate the F1 value of each label, and then average it as the final indicator. The method is called Macro-F1 and can reduce the impact of too many “O” labels.

#### 4.1.2. Implementation Details

**Figure 4:**
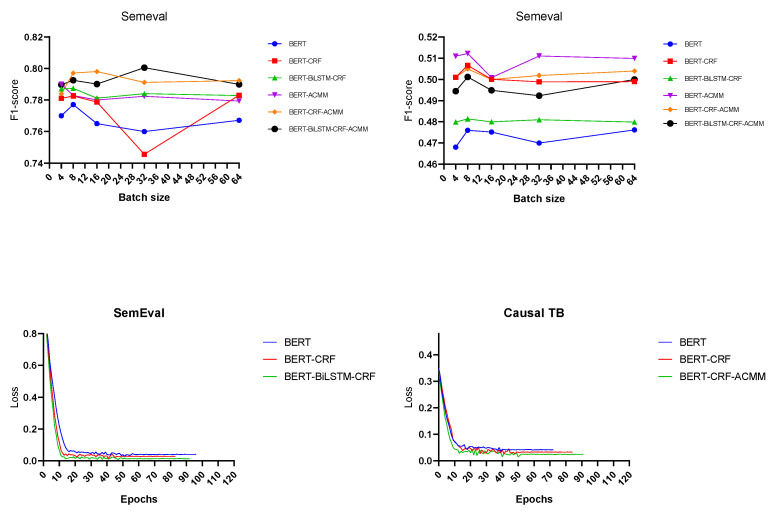
Experimental diagram of hyperparameter selection. The two pictures above show the results of models under different batch sizes. The remaining two pictures show the loss curve of the model.

We adopt mini-bach mechanism to train our model. As seen in the two pictures above, in Figure 4, we have conducted many experiments by setting the batch size to [4,8,16,32,64], and, finally, we have chosen a better batch size of 8. As for the learning rate, we found that it is a better choice to choose different learning rates for different parameters through experiments. We set the learning rate of the $\theta_{encoder}$ parameters to $1\times 10^{-5}$, and the learning rate of the parameters of $\theta_{a2c}$ to $1\times 10^{-4}$. As for the learning rates of the models BiLSTM [34] and TARGER [35], we refer to the corresponding values in the paper, which are 0.1 and 0.01, respectively.

We first use 10 epochs hot start to fine-tune the encoder model. As shown in the last two pictures of Figure 4, the model to be stabilize at about 10–15 epochs. In order to choose a more stable language model, we think the model in 10 epochs is a better choice. We also use an early stopping mechanism, and the training is terminated when the performance on validation set has not improved in the last 20 epochs. We use the Adam optimizer for training, and the parameters selected the recommended options in the paper [36].

### 4.2. Baselines

To illustrate the effect of our method in the causal event extraction task, we choose both BiLSTM-based and BERT-based methods that achieve state-of-the-arts results as baselines in our experiments.

**BiLSTM-CRF** [34] is a classic sequence labeling method, including a BiLSTM encoder and a CRF classifier.

**TARGER** [35] is a sequence labeling model that uses CRF classifier and CNN-BiLSTM encoder to learn Char-level features and Word level features.

**BERT** [17] is a large pre-trained language model composed of multiple transformer blocks, performing well in multiple downstream tasks.

**BERT-CRF** and **BERT-BiLSTM-CRF** are extensions of the BERT model, using pre-trained word embedding generated by BERT as input.

BERT*-ACMM is our proposed model, among which ACMM (Actor-Critic Mask Model) is a model for confrontation with the BERT model and extensions of BERT model. All the parameters of the above BERT model use the huggingface’s bert-base-uncased version (https://huggingface.co/models, accessed on 13 March 2020).

### 4.3. Experimental Results

In Table 2, we report the performance of the baseline and our model on the task of causality extraction. The first part of the table is the traditional model that does not use the BERT pre-trained model, the second part is the model that uses BERT as encoder, and the third part is the second part of the model with our adversarial model components.

In the first part of the Table 2, we chose traditional neural network models, such as CNN and RNN. The experimental results show that on this small dataset, the generalization performance of the model is not good. As a recently popular pre-training model in NLP, we use BERT and its variants as a representative of this type of model. The experimental results show that this model is better than the traditional network structure. There are 12 points and 3 points of F1-value improvement on SemEval and Causal TB, respectively. This is due to its large amount of language pre-training knowledge and complex structure. However, this improvement is not endless. On the smaller dataset Causal TB, we can see that, due to the increase in model complexity, its generalization is weakened. From BERT-CRF to BERT-BiLSTM-CRF, the F1-value of the model is reduced by 2.52.

Since our model only interacts with the language model at word granularity, it does not increase its model complexity. However, after adding our adversarial component to each model, its performance has improved. For example, the BERT model has increased the F1 value by 0.57 and 3.63, respectively. Similarly, the boost values of the BERT-BiLSTM-CRF model on the two datasets are 0.51 and 1.98. This is very exciting because our adversarial component works better on small datasets (Causal TB). These experiments show that, when increasing model complexity is no longer effective, we provide an alternative method to continue to improve model performance.

### 4.4. Analysis of Different Mask Strategy

In this section, we compared the masking strategy we generated with various rule-based masking strategies. The following is a detailed introduction of each word masking method:

**No Mask** uses the basic language model (BERT) and linear classifier for fine-tuning without using any mask strategy.

**Whole Word** takes the entire word as a unit and randomly mask a specified number of words [37].

**Word Piece** is used during BERT pre-training. For the word sequence that has been segmented, randomly mask the specified proportion of tokens.

**Span** randomly selects multiple consecutive tokens for masking for each sentence [19].

As shown in Table 3, we used three random word masking strategies to compare with our strategy. It can be seen that, although this data enhancement method has obvious effects on the Causal TB dataset, it is not effective for the SemEval dataset. It is nearly 3 points in Causal TB, and not improved in SemEval. This result shows that, in large datasets, this data augmentation method is not particularly effective. The reason is that, as many recent studies have shown, this simple noise is limited to the improvement of the model [38]. However, our reinforcement learning strategy is different from it. We use the propagation of loss to generate difficult samples to obtain better results. As you can see, our strategy has achieved the best results compared to other strategies. Even in the SemEval dataset, the best recall appears in the Whole Word strategy, but its precision is significantly reduced. This causes its F1 value to be inferior to our strategy, the same as Causal TB.

**Figure 5:**
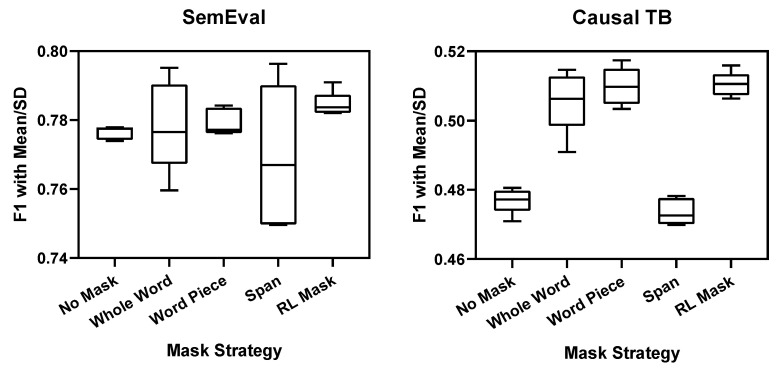
Box plot of F1 for 5 different strategies. The upper and lower sides represent the maximum and minimum values, and the middle line represents the mean values.

At the same time, another point that shows our model is superior to rule-based strategies is its stability. To demonstrate this, we performed 5 repeated experiments for each model using random training seeds. As shown in Figure 5, we use box plots to show the mean and variance of various strategies. It can be seen that the variance of the model without any masking strategy is the smallest. When the random strategy is adopted, the variance of the model becomes larger. This is because, in addition to the randomness of the model parameters, the randomness of the masking words is added. This result makes the model more unstable, and the result of each experiment depends on the random result. However, as can be seen from the figure, although our model adds randomness, it is more stable. This is because our strategy is based on the probability distribution $p_{a}$ output by the ACMM model, which has a higher probability of deleting important words. This kind of method can not only generate difficult samples but also ensure stability.

### 4.5. Analysis of Masked Sentence

**Figure 6:**
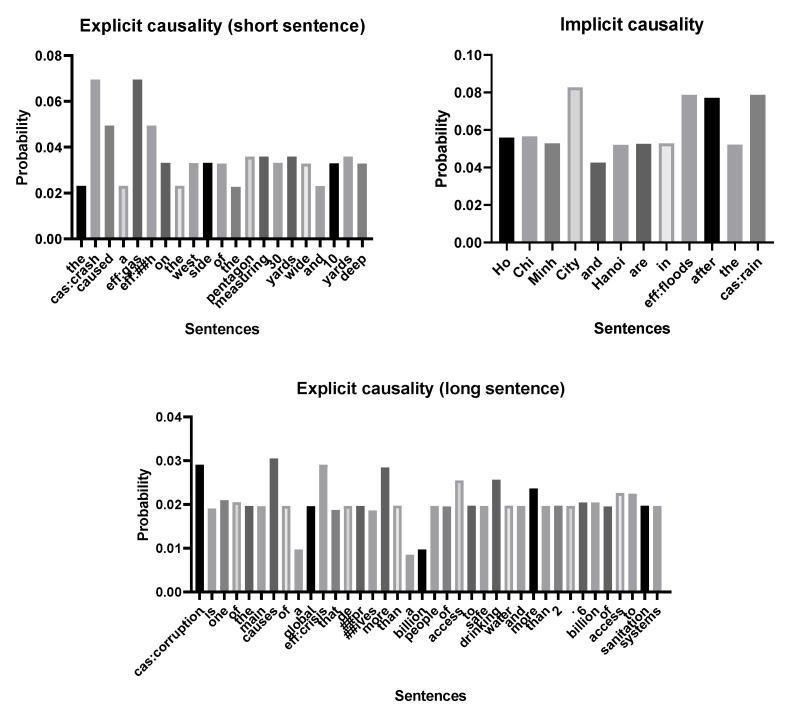
Examples of three masked sentences. Among them, “cas” represents the cause entity, and “eff” represents the result entity. There are two sentences with explicit causality, and one sentence with implicit causality.

In Figure 6, we have selected three sentences to show the effect of the mask model. It can be seen that, whether it is explicit causality or implicit causality, the causal entity and the words in the causal entity always have a higher probability of being masked (For the stability of the model, we adopted some heuristics, that is, the causal entity is not masked.). The possible reason is that obliterating causal entities is the most likely to reduce the accuracy of model, and the words between entities also have a high probability of indicating whether the entities have causal relationships.

In explicit causality, the model can accurately distinguish causal connectives whether in long sentences or short sentences. In implicit causality, although the word between the entity does not clearly indicate causal relation (e.g., “after”), it is more likely to cause prediction errors in the model than other words in the sentence. From the perspective of information theory, our model is more willing to delete words that contain more information for predicting causality. For example, our concealment probability for meaningless conjunctions (such as “a”, “the”, and “of”) is much lower than words with actual meaning (such as “city”, “pentagon”, “yards”, etc.).

## 5. Conclusions

We propose a novel framework that can automatically generate adversarial attacks on language models at word granularity. The adversarial attacks we generate for causality extraction tasks can make the model achieve better generalization performance on small datasets. To this end, we use the actor-critic model to generate adversarial examples in a targeted manner. We performed empirical studies on multiple models and different rule-based mask strategies, which show the effectiveness of our optimal mask strategy.

## Figures and Tables

**Table 1:** Experiment dataset statistics. ***Cause-effect*** means that sentence contains causal-effect pairs.

	SemEval		Causal TB	
	Train	Test	Train	Test
***Cause-effect***	904	421	220	181
***Other***	6574	2775	202	78
***All***	7478	3196	422	103

**Table 2:** Results on two causal extraction datasets.

Model	SemEval			Causal TB		
	P	R	F1	P	R	F1
BiLSTM-CRF	65.90	45.37	53.72	32.07	31.41	31.64
TARGER	61.38	70.20	65.49	40.69	49.55	44.69
BERT	77.61	77.85	77.71	44.01	53.85	47.60
BERT-CRF	78.63	77.91	78.26	55.24	46.80	50.66
BERT-BiLSTM-CRF	80.40	77.20	78.74	54.60	43.60	48.14
BERT-ACMM	79.19	77.41	78.28	48.37	**54.49**	**51.23**
BERT-CRF-ACMM	80.19	**79.23**	**79.71**	54.33	47.18	50.50
BERT-BiLSTM-CRF-ACMM	**81.55**	77.08	79.25	**55.36**	45.78	50.12

**Table 3:** Comparison of our method with rule-based strategies.

Mask	SemEval			Causal TB		
Function	P	R	F1	P	R	F1
No Mask	77.61	77.85	77.71	44.01	53.85	47.60
Whole Word	76.19	**79.92**	77.99	48.38	53.85	50.94
Word Piece	76.54	78.67	77.58	**52.31**	49.36	50.78
Span	77.04	76.13	76.58	43.13	53.85	47.90
RL Mask	**79.19**	77.41	**78.28**	48.37	**54.49**	**51.23**

## Data Availability

Publicly available datasets were analyzed in this study. This data can be found here: https://github.com/zy234/ACMM/tree/main/data, accessed on 15 June 2021.

## Abbreviations

The following abbreviations are used in this manuscript:

ACMM	Actor-Critic Mask Model
SOTA	state of the art
A2C	Advantage Actor-Critic
MLM	Mask Language Model
NLP	Natural Language Processing
DQN	Deep Q-Learning
Causal TB	Causal TimeBank
TD	Temporal-Difference
